# Nanobolometer with ultralow noise equivalent power

**DOI:** 10.1038/s42005-019-0225-6

**Published:** 2019

**Authors:** Roope Kokkoniemi, Joonas Govenius, Visa Vesterinen, Russell E. Lake, András M. Gunyhó, Kuan Y. Tan, Slawomir Simbierowicz, Leif Grönberg, Janne Lehtinen, Mika Prunnila, Juha Hassel, Antti Lamminen, Olli-Pentti Saira, Mikko Möttönen

**Affiliations:** 1 QCD Labs, QTF Centre of Excellence, Department of Applied Physics, Aalto University, P.O. Box 13500, 00076 Aalto, Finland.; 2 VTT Technical Research Centre of Finland Ltd, QTF Centre of Excellence, P.O. Box 1000, 02044 VTT, Finland.; 3 National Institute of Standards and Technology, Boulder, CO 80305, USA.; 4 Department of Applied Physics, California Institute of Technology, MC 149-33, Pasadena, CA 91125, USA.

## Abstract

Since the introduction of bolometers more than a century ago, they have been used in various applications ranging from chemical sensors, consumer electronics, and security to particle physics and astronomy. However, faster bolometers with lower noise are of great interest from the fundamental point of view and to find new use-cases for this versatile concept. We demonstrate a nanobolometer that exhibits roughly an order of magnitude lower noise equivalent power, $20\text{zW}/\sqrt{\text{Hz}}$, than previously reported for any bolometer. Importantly, it is more than an order of magnitude faster than other low-noise bolometers, with a time constant of 30 *μ*s at $60\text{zW}/\sqrt{\text{Hz}}$. These results suggest a calorimetric energy resolution of 0.3zJ=h×0.4THz with a time constant of 30 *μ*s. Further development of this nanobolometer may render it a promising candidate for future applications requiring extremely low noise and high speed such as those in quantum technology and terahertz photon counting.

Bolometry is one of the oldest radiation-sensing techniques^1^ dating back to 1880. Yet, it remains competitive and widespread^2–5^, mainly owing to the flexibility bolometers offer in terms of the center frequency, bandwidth, and dynamic range, as well as the possibility of energy-resolving calorimetric operation^6^. These properties render bolometers, devices that detect radiation-generated heat in an absorber, interesting for the emerging quantum technological systems such as superconducting quantum computers^7,8^. Bolometers could, for example, be used as broadband measurement devices^9^ for solid-state or flying qubits^10^ and for characterization of cryogenic environments, cabling, and microwave components^11–16^ at powers in the single-photon regime. The quantum technological devices operate at such ultralow powers, and consequently call for characterization and calibration in the single-photon regime.

Very recently, Opremcak et al.^17^ demonstrated a readout scheme for superconducting qubits where a microwave bolometer may appear useful in bringing flexibility for the frequency band of the readout signal. Although future development of the method and qubits may bring a relief on the speed (≲1 μs time constant) and energy resolution (≲100 yJ) requirements realized by Opremcak et al.^17^, this potential application calls for improvements on the state-of-the-art fast ultralow-noise bolometers.

Outside quantum technology, bolometers are widely utilized by astronomers in both ground^18^ and space-based observatories^19,20^, and in other types of detectors^21,22^. Although this is a mature field requiring relatively high technology readiness levels and scalability for the detectors, some of the future applications call for improvements in the intrinsic noise of the existing bolometers. Interestingly, microwave bolometers have recently emerged as a potential candidate for the detection element in the dark matter experiments^23–25^, where the bolometer could either be used to directly measure the extremely low powers arising from photons generated in axion haloscopes^26^ or they could be applied to detect dark matter-generated quasiparticles that diffuse into the bolometer. In the former case, the advantage of bolometers over microwave amplifiers is that they are resilient to quantum fluctuations, but further development in the bolometer noise level is needed to achieve reasonable integration times in the experiments.

The noise equivalent power (NEP) quantifies the input power resolution of the bolometer in a unit bandwidth. Relatively recently, thermal conductances below 1 fW/K between bolometers and their environment have been measured^27,28^, implying that the lower bound for NEP set by thermal energy fluctuations can be reduced down to at least $10\text{zW}/\sqrt{\text{Hz}}$. To date, however, the lowest measured NEPs for bolometers are around $300\text{zW}/\sqrt{\text{Hz}}$^29,30^, achieved using transition edge sensors^31^ (TESs) in far-infrared regime. For example, the expected power level to be detected in the Axion Dark Matter Experiment^32^ is of the order of 10^−22^ W. With the current state-of-the-art bolometers, the integration time to reach unit signal-to-noise ratio is of an order of a thousand hours. Thus, lowering noise of bolometers to $10\text{zW}/\sqrt{\text{Hz}}$ would bring the integration time to the reasonable single-hour time scale.

Promising NEPs have also been reported in the terahertz range for alternative device concepts, such as kinetic inductance detectors (KIDs) $\left(400\text{zW}/\sqrt{\text{Hz}}\right)$^33^ and proof-of-principle quantum-capacitance detectors (of order $10\text{zW}/\sqrt{\text{Hz}}$)^34,35^. Both of these detect radiation-generated non-equilibrium quasiparticles in a superconductor. In addition, the background rate of the random telegraph noise in semiconducting charge sensors shows potential for extremely low NEP $\left(1\text{zW}/\sqrt{\text{Hz}}\right)$^36,37^ in the terahertz regime. However, the coupling efficiency to a radiation source is expected to be low and full experimental characterization of the efficiency has not been reported.

We recently introduced a threshold microwave detector based on superconductor–normal-metal–superconductor (SNS) junctions^38^ and showed that it can detect weak microwave pulses down to the zeptojoule level in a time-gated detection mode^28^. In this work, in contrast, we implement a continuously operating SNS bolometer. We measure an $\text{NEP}\approx 50\text{zW}/\sqrt{\text{Hz}}$ with a time constant of 0.6 ms. We can tune the time constant, in situ, below 100 μs at the expense of increasing the NEP to $80\text{zW}/\sqrt{\text{Hz}}$, which is nevertheless still lower than the lowest previously reported bolometer NEPs^29,30^. By introducing a Josephson parametric amplifier^39^ (JPA) to the bolometer readout circuit, we further reduce the NEP to a record low value of $20\text{zW}/\sqrt{\text{Hz}}$. For an NEP of $60\text{zW}/\sqrt{\text{Hz}}$, we achieve response times down to about 30 μs using the JPA, which is one to two orders of magnitude faster than those reported for the most sensitive TESs^30^. Although the experiments described here focus on the measurement of the input power, the achieved NEP and time constant suggest that, in a calorimetric mode of operation, the energy resolution may be well below the current 1.1-zJ record for thermal detectors^28^. Furthermore, we study the feasibility of the SNS bolometer in the terahertz regime by simulating a silicon lens with double-slot terahertz antenna coupled to the bolometer. Our results suggest a single-photon detection rate of 100 photons per second at 1.3 THz from a 3-K thermal source.

## Results

### Device and measurement setup.

**Fig. 1:**
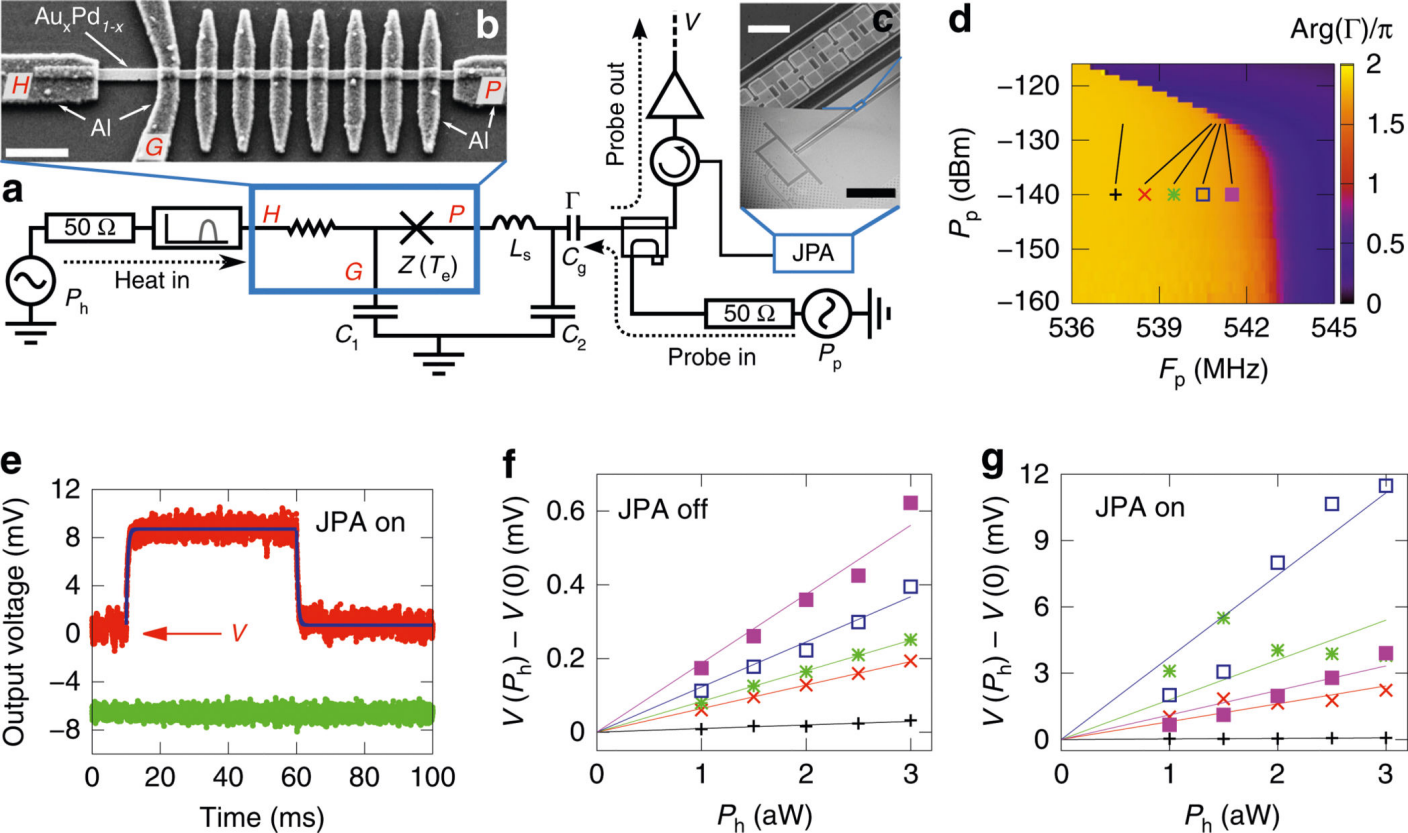
Measurement setup and device characterization. **a–c** Simplified detector measurement setup (**a**) together with micrographs of the superconductor–normal-metal–superconductor (SNS) junctions between leads H, G, and P taken from a similar device (**b**) and of a similar Josephson parametric amplifier (JPA) (**c**). The impedance $Z\left(T_{\text{e}}\right)$ of the series of short SNS junctions forms a temperature-sensitive resonant circuit together with a meander inductor $L_{\text{s}}\approx 1.2\;\text{nH}$ and the capacitors $C_{1}\approx 87\;\text{pF}$ and $C_{2}\approx 33\;\text{pF}$. The gate capacitance is $C_{g}\approx 0.87\;\text{pF}$. There is an 8.4-GHz bandpass filter connected to lead H. The scale bar in **b** indicates 1 μm and in **c** 15 μm (top) and 400 μm (bottom). **d** Phase of the reflection coefficient at the gate capacitor Γ as a function of probe frequency $f_{\text{p}}$ and power $P_{\text{p}}$, without heating and with the JPA off. **e** Example of the ensemble-averaged detector output voltage at the digitizer V (top curve) and voltage in the other quadrature (bottom curve), with the JPA on. The blue curves show exponential fits to the rising and falling edges of the signal. **f, g** Change in the detector output voltage at the digitizer after the heater is turned on (markers, see **e**) as a function of the finite heater power $P_{\text{h}}$ with the JPA off (**f**) and on (**g**). The typical error of voltage measurement is of the order of size of the symbols. The $\left(f_{\text{p}},P_{\text{p}}\right)$ operation points are indicated in **d**. The JPA shifts the resonance frequency slightly, thus the highest output is found at slightly different operating point. The bath temperature is $T_{\text{b}}=25\;\text{mK}$ for all data in this paper

Figure 1a, b shows the detector and its measurement scheme. We couple the detector to an 8.4-GHz microwave source through a 50-Ω transmission line, which allows us to calibrate the heater power $P_{\text{h}}$ incident at the detector input with a decibel level of uncertainty. Essentially all incident heater power is absorbed by the long SNS junction between the leads H and G since the junction is long enough for its impedance to be almost entirely real, 36 Ω, and well matched to the transmission line impedance of $Z_{0}=50\;\text{Ω}$. The imaginary part of the impedance, arising from the capacitor $C_{1}$, is roughly −i0.2Ω and an order of an ohm from parasitic series inductance at 8.4 GHz, and can be therefore neglected. Thus, an increase in $P_{\text{h}}$ leads to an increase in the electron temperature $T_{\text{e}}$ in the ${\text{Au}}_{x}{\text{Pd}}_{1-x}(x\approx 0.6)$ nanowire used as the normal-metal part in the SNS junctions. This in turn results in an increased inductance of the short SNS junctions^40,41^ between leads P and G, which implies a lower resonance frequency of the effective LC oscillator formed by the short SNS junctions, the on-chip meander inductor $L_{\text{s}}$, and the on-chip parallel plate capacitors $C_{1}$ and $C_{2}$. We detect this change by measuring the reflection coefficient at the detector gate capacitor $\text{Γ}\left(T_{\text{e}},\;\omega_{\text{p}}\right)$ at a fixed probe frequency $f_{\text{p}}=\omega_{\text{p}}/2\pi$. See Methods for the extraction of Γ from the detector output voltage at the digitizer V. Furthermore, we have the option to amplify the readout signal with a JPA^42^ (Fig. 1c, see Methods).

### Characterization experiments.

Figure 1d shows the phase of the reflection coefficient at the gate capacitor (see Methods for details) as a function of probe frequency and probe power, at zero heater power. The most striking feature in Fig. 1d is the decreasing resonance frequency as the probe tone begins to significantly heat the electrons in the SNS junctions above $P_{\text{p}}=-135\;\text{dBm}$. This electrothermal feedback^43^ can be used to optimize the sensitivity and the time constant of the detector or even induce temperature bistability^28^ (not visible in Fig. 1d).

The NEP is determined by how noisy the readout signal is relative to the responsivity of the signal to changes in $P_{\text{h}}$ (see Methods). Thus in Fig. 1e, we show an example of the detector output voltage V, which is defined as the voltage in the quadrature providing the greatest response to the heater power after amplification (≈103 dB), demodulation, and an optimally chosen phase rotation. In Fig. 1e, we first set $P_{\text{h}}$ to a small value (3 aW) for a period of several tens of milliseconds, then turn $P_{\text{h}}$ off for a similar period, and finally average over repetitions of this modulation pattern. From such data we extract the quasistatic voltage response at the digitizer and the time constant using exponential fitting functions. Figure 1f, g shows the quasistatic response of the detector output voltage to the heater power up to 3 aW. We define the detector responsivity as the ratio of the voltage response and the corresponding heater power. We also employ this information to choose an appropriate power level for the heater in our experiments discussed below.

### Dimensionless susceptibility.

To understand the detector response at high probe power, we develop a model for the electrothermal feedback^28,44^ (see Methods for details). We define a dimensionless susceptibility as

(1) $$\chi ={\frac{\partial \text{Δ}P}{\partial P_{\text{h}}}|}_{\partial_{t}\text{Δ}P=0},$$

where ΔP equals the amount of additional heat flowing from the nanowire electrons to their thermal bath. Therefore, χ is the factor by which the probe-induced electrothermal feedback enhances the heating of the bolometer relative to the externally applied power $P_{\text{h}}$.

### Detector responsivity and noise.

**Fig. 2:**
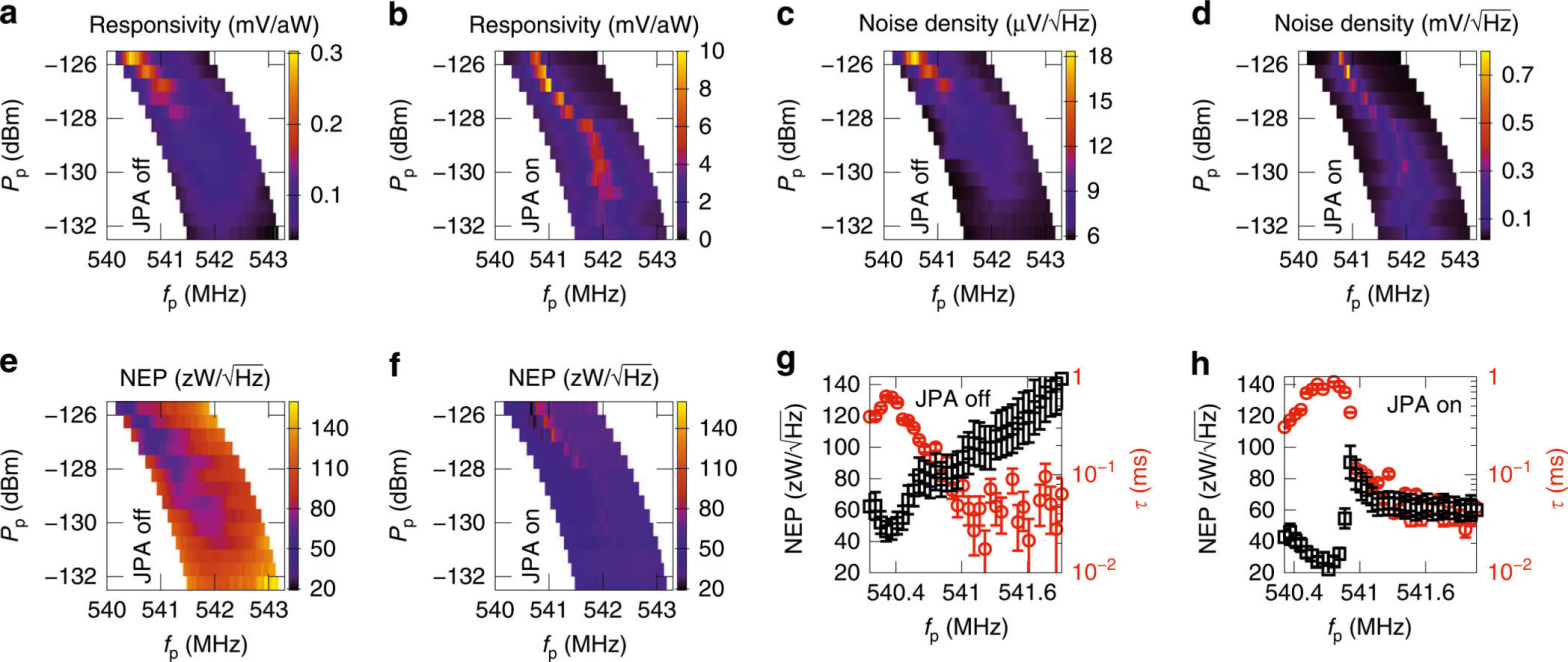
Noise equivalent power (NEP) and thermal time constant. **a–d** Quasistatic responsivity of the probe voltage to the heater power (**a, b**) and probe voltage spectral density (**c, d**) as functions of probe frequency and power with the Josephson parametric amplifier (JPA) off (**a, c**) and on (**b, d**) averaged over noise frequencies between 20 and 100 Hz. **e, f** Noise equivalent power as a function of probe frequency and power with the JPA off (**e**) and on (**f**) averaged over noise frequencies between 20 and 100 Hz. **g, h** NEP (black) and thermal time constant (red) with the JPA off at fixed $P_{\text{p}}=-126\;\text{dBm}$ (**g**) and with the JPA on at $P_{\text{p}}=-126.5\;\text{dBm}$ (**h**). The error bars indicate the standard error of the mean for the time constant and error arising from heater power calibration for NEP

In Fig. 2a, b, we show the responsivity of the detector output voltage. Note that the NEP is unaffected by the calibration of the gain of the readout circuitry since the measured responsivity and noise are both amplified equally. The responsivity is maximized for probe frequencies close to the resonance. As the probe power is increased, the width of the resonance decreases, leading to a sharp increase in the responsivity. Note that the color scales are different in Fig. 2a, b, since the JPA adds gain in excess of 20 dB.

Interleaved with the measurements of the responsivity, we also record separate noise spectra for the detector output voltage and for the out-of-phase quadrature at each probe power and frequency. In Fig. 2c, d, we show the voltage noise spectral density across the same range of $f_{\text{p}}$ and $P_{\text{p}}$ as for the responsivity. Let us first discuss the low-probe-power limit $\left(P_{\text{p}}≲-132\;\text{dBm}\right)$ with the JPA off in Fig. 2c. In this case, the electrothermal feedback is negligible (χ≈1), ΔP vanishes, and the spectrum is dominated by noise added by the amplifiers in the readout circuitry. Furthermore, the noise power assumes a similar value on and off resonance. However, with the JPA on in Fig. 2d, we consistently observe a peak in the noise near the resonant probe frequency, indicating that amplifier noise is not dominating the signal even at the lowest probe powers shown (−132.5 dB). With the JPA off, the thermal fluctuations of the detector surpass the amplifier noise only at high $P_{\text{p}}$.

### NEP and time constant.

Figure 2e, f presents the main results of this paper, that is, the measured NEP for an 8.4-GHz input over a range of probe powers and frequencies. We compute the NEP by dividing the voltage spectral density by the quasistatic responsivity and multiplying the result by a factor $\sqrt{1+{\left(2\pi \tau f_{\text{n}}\right)}^{2}}$, where $f_{\text{n}}$ is the noise frequency. This factor takes into account the fact that the thermal time constant τ decreases the responsivity of the detector with respect to the quasistatic case (see Methods). Figure 2e, f shows the NEP with the JPA on and off, respectively, averaged over noise frequencies from 20 to 100 Hz.

In Fig. 2g we show the NEP and the time constant as functions of the probe frequency at fixed $P_{\text{p}}=-126\;\text{dBm}$ with the JPA off. Figure 2h is measured in identical conditions except that the JPA is on and the probe power is set to −126.5 dBm. The electrothermal feedback is strong and positive (χ≫1) at probe frequencies just below the resonance frequency. In contrast, the electrothermal feedback is strongly negative (χ≪1) at probe frequencies just above the resonance. This is clearly visible in the time constant $\tau =\chi \tau_{\text{b}}$, which increases by nearly an order of magnitude as the probe approaches the resonance despite the fact that the bare thermal time constant $\tau_{\text{b}}$ simultaneously decreases owing to increased electron temperature. Here, $\tau_{\text{b}}$ denotes the time constant in the absence of electrothermal feedback (see Methods). The lowest NEP of $20\text{zW}/\sqrt{\text{Hz}}$ in Fig. 2h coincides with the peak of the time constant (1 ms), suggesting that at $P_{\text{p}}=-126.5\;\text{dBm}$ the NEP is optimized at the frequency that maximizes χ.

As the probe frequency exceeds the resonance, the time constant quickly decreases by more than an order of magnitude below 100 μs. In this regime, the positive effect of the JPA is particularly clear: the NEP degrades quickly with increasing probe frequency if the JPA is disabled, but stays roughly constant when it is enabled. The fact that the NEP remains relatively flat at $60\text{zW}/\sqrt{\text{Hz}}$ with the JPA on (Fig. 2h) is an indication that the internal fluctuations of the detector are limiting the performance instead of amplifier noise. This is an example of the convenient in situ tunability of the SNS detector, that is, we can choose a different trade off between the NEP and the time constant by a small change of the probe frequency or power. We can also tune the time constant and the dynamic range by changing the bath temperature or by applying an additional constant heating power through the heater port. However, such an optimization is left for future work.

### Noise analysis.

**Fig. 3:**
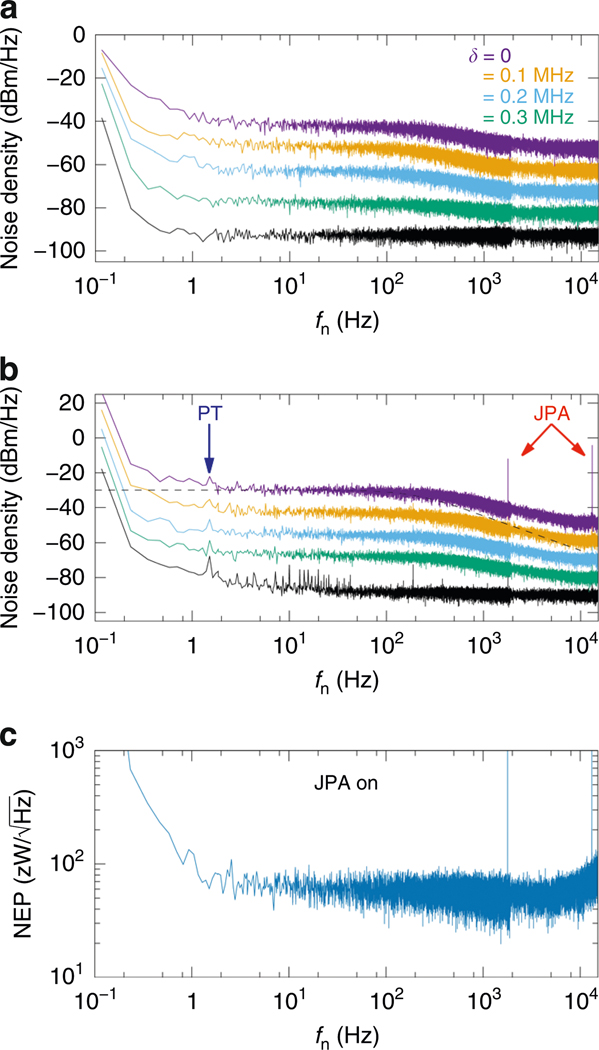
Frequency spectra of voltage noise and noise equivalent power (NEP). **a, b** Spectral density of the noise in the signal quadrature of the down-converted probe tone with the Josephson parametric amplifier (JPA) off (**a**) and on (**b**) as functions of the noise frequency $f_{\text{n}}$. The bottommost curve shows the spectral density far off resonance at $f_{\text{p}}=539.275\;\text{MHz}$ and $P_{p}=-132.5\;\text{dBm}$, whereas the green, blue, orange, and yellow curves are measured at $P_{\text{p}}=-126\;\text{dBm}$ (JPA off) and $P_{\text{p}}=-126.5\;\text{dBm}$ (JPA on), and span a narrow frequency range near the resonance. Specifically, the probe frequencies are $f_{\text{p}}=540.6125\;\text{MHz}-\delta$, where the values of δ are indicated in **a**. For clarity, the curves have been offset vertically in increments of 10 dBm/Hz. The two peaks above 1 kHz in **b** are due to the aliased JPA idler. The excess noise at multiples of 1.4 Hz is attributed to the pulse tube (PT) cryocooler. The dashed line indicates a first-order RC filter response with a time constant identical to that in the δ=0 trace in **b**. **c** NEP with the JPA on as a function of the noise frequency at $P_{p}=-126.5\;\text{dBm}$ and $f_{\text{p}}=541.9625\;\text{MHz}$. These data yield 0.3 zJ for the energy resolution estimate of the detector (see text). Discontinuity in the data on all panels near 2 kHz is caused by the fact that we measure the high- and low-frequency noise separately with different time steps

In Fig. 3a, b, we present the full noise spectrum of the output signal at $P_{\text{p}}=-126\;\text{dBm}$ and $P_{\text{p}}=-126.5\;\text{dBm}$ with the JPA off and on, respectively. Above 1 Hz and below 1 kHz, the noise increases up to 14.5 dB above the broadband background set by the amplifier noise for probe frequencies near resonance. Far off-resonance we find only the broadband amplifier noise floor in addition to $1/f_{\text{n}}$ noise. We also observe in Fig. 3b noise peaks at multiples of 1.4 Hz, matching the frequency of vibrations caused by the pulse tube cryocooler. Note that these peaks are clearly visible only when the JPA is on and the probe is far from the resonance, suggesting that the pulse tube noise does not couple directly to the detector, but rather to the amplifiers. At operation points with low NEP, the pulse tube noise is masked by the noise generated by the detector itself.

### Predicted energy resolution.

In Fig. 3c, we present the NEP measured with the JPA on as a function of the noise frequency at a $\left(f_{\text{p}},P_{\text{p}}\right)$ point selected for short time constant and low NEP. From the NEP, we can obtain an estimate for an upper bound on the energy resolution^45^

$$\varepsilon \approx {\left(\int_{0}^{\infty }\frac{4\text{d}f}{\text{NEP}{\left(f\right)}^{2}}\right)}^{-1/2}.$$

By restricting the above frequency integration below the thermal cutoff frequency 1/(2πτ)=5.8kHz, the data in Fig. 3c yields ε=0.32zJ=h×480GHz, surpassing, for example, the anticipated resolution of the TES-based Fourier transform spectrometer^19^ specified to have about an octave of resolution in the band of 1.4–9 THz. Increasing the cutoff frequency to 10 kHz yields ε=0.26zJ=h×390GHz. Here, h denotes the Planck’s constant.

### Feasibility for terahertz detection.

**Fig. 4:**
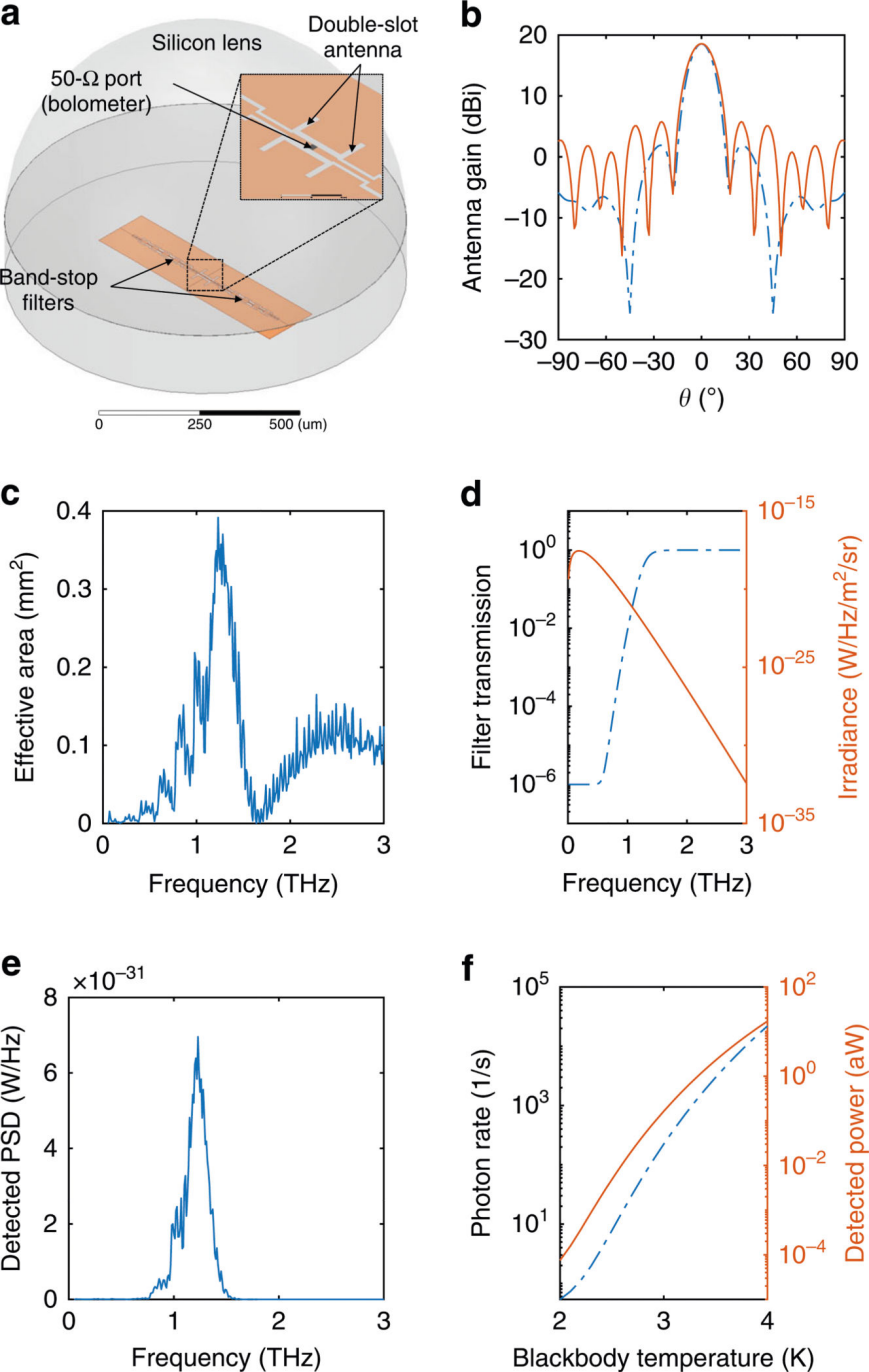
Terahertz antenna and detection simulations. **a** Electromagnetic simulation model of an extended hemispherical silicon lens with a double-slot antenna as a feed. **b** Antenna gain at 1.3 THz as a function of the incident angle in the two principal planes, H-plane (dashed line) and E-plane (solid line). The H-plane (E-plane) is the plane containing the magnetic (electric)-field vector and the direction of maximum radiation^49^. **c** Effective detection area as a function of frequency. **d** Planck spectral irradiance at T=3.0K (solid line), and projected frequency-selective surface (FSS) filter transmission (dashed line) for blackbody measurements. **e** Computed photon rate power spectral density (PSD) corresponding to **d**. **f** Computed total detected photon rate (dashed line) and the corresponding detected power (solid line) as a function of the temperature of the blackbody emitter

Inspired by the above-suggested energy resolution, we theoretically study the future feasibility of the SNS bolometer as a THz detector. We simulate a complete experiment, including a possible THz antenna design, aimed at detecting individual photons from a thermal source. The THz coupling scheme is based on a substrate-lens-coupled planar antenna. An extended hemispherical silicon lens (diameter 1 mm) integrated with a double-slot antenna as a feed^46^ was designed for the center frequency of 1.3 THz (see Fig. 4a). We employ electromagnetic simulations to study the performance in THz detection. Note that at the considered signal frequency range, well exceeding the Bardeen–Cooper–Schrieffer gap frequency, even a fully superconducting bolometer acts as a resistive load, and therefore no separate load resistor is needed. Thus, for simplicity, the bolometer is modeled as a 50-Ω port in the simulations. We have designed band-stop filters at 1.3 THz to prevent the bolometer readout circuitry from interfering with the antenna^47^. The radiation patterns at 1.3 THz (Fig. 4b) show −3-dB beam widths of about 8°. The detector efficiency is quantified by the effective area $A_{\text{e}}$, peaking to 0.35 mm^2^ at 1.3 THz (Fig. 4c).

To analyze the detected power and photon count rates, we assume a blackbody thermal source represented by the Planck spectral irradiance for a single polarity expressed as $B_{\text{r}}(f)=(hf^{3}/c^{2})/\left\{\exp\left[hf/\left(k_{\text{B}}T\right)\right]-1\right\}$, where $k_{\text{B}}$ is the Boltzmann constant, T is the temperature of the source, and c is the speed of the light. We aim at a detection band above the peak frequency at $hf∼k_{\text{B}}T$. Thus, we must filter the low-frequency tail very efficiently, as it represents orders of magnitude higher power density in comparison to the band of interest.

Such filtering between the blackbody source and the detector can be achieved, for example, by metal mesh structures acting as frequency-selective surfaces (FSSs)^48^. Filter systems based on FSS structures have been demonstrated previously in the context of low-power detector experiments: de Visser et al.^33^ employ a bandpass-filter system with a low-frequency roll-off above 60 dB per octave, and a stop-band rejection of more than 60 dB. For our purposes, we aim to capture the essential features of such filters by defining a high-pass filter with a comparable roll-off, stop-band transmission of −60 dB, and a 3-dB cutoff at 1.3 THz.

The obtained frequency response η(f) is shown in Fig. 4d, along with the Planck spectral irradiance for T=3.0K. From these data, the detected power spectral density is obtained as $S(f)=\int \eta B_{r}A_{\text{e}}\text{Ωd}f$, where Ω is the solid angle corresponding to the antenna beam width. Taking the optical throughput $A_{\text{e}}\text{Ω}$ from the antenna simulations of Fig. 4b, we show the spectral density of the detected photon count per unit bandwidth S(f)/(hf) in Fig. 4e. The total detected power and photon count are shown in Fig. 4f as functions of the blackbody temperature. In the vicinity of the blackbody temperature of T≈3K, the power level is well within the sensitivity range of the detector, and the photon rates are compatible with the speed of the detector. Thus, this seems a possible scenario to demonstrate terahertz photon counting in the future using the SNS detector.

## Discussion

Our demonstration of an NEP of $20\text{zW}/\sqrt{\text{Hz}}$ for an SNS junction-based bolometer is an order of magnitude improvement over results reported for TESs and KIDs. In this sensitivity range, we also report response times down to 30 μs, that is, detector bandwidth of about 5 kHz. For comparison, the most sensitive TESs and KIDs operate at bandwidths of order 100 Hz^19,33^. The observed detector speed and NEP predict an energy resolution compatible with single-photon detection extending down to the low terahertz range, ~400 GHz.

Recently, superconducting qubits have shown great progress in detecting single microwave photons^50–54^, but this technology is currently limited to frequencies below 10 GHz. Thus, the SNS detector provides a valuable complementary approach for future research.

Accommodating the SNS detector with a terahertz antenna in the future is supported by our simulations of an experimental detection scheme in the terahertz regime. Note, that the impedance of the SNS detector may be varied by the choice of the normal-metal size and aspect ratios thus providing flexibility in impedance matching. Furthermore, the radio frequency (rf) readout of SNS detectors is naturally suited for frequency multiplexing^19,55,56^. Thus, future development of the SNS detector may render it a potential candidate for various applications.

The design of the SNS detector is naturally compatible with the integration of superconductor–insulator–normal-metal junctions to the absorber^57^. This may allow direct cooling of the normal-metal electrons, and thus a possibility to increase the dynamic range of the detector using feedback. We estimate the achievable cooling power, and hence the dynamic range, to be well in the pW range^57^.

## Methods

### Sample fabrication and measurement setup.

For details of the sample fabrication methods and of the measurement setup, see Govenius et al.^28^.

### Reflection coefficient.

Before feeding the probe signal into the cryostat, we split a fraction of it into a reference tone. We digitize both signals, the reference and the eventual probe signal, which is reflected from the detector gate and subsequently amplified and guided out of the cryostat. We define $\tilde{\text{Γ}}\left(T_{\text{e}},\omega_{\text{p}}\right)$ as the ratio of the reflected signal and the reference signal. We obtain the reflection coefficient at the gate capacitor $\text{Γ}\left(T_{\text{e}},\omega_{\text{p}}\right)$ by first measuring $\tilde{\text{Γ}}\left(T_{\text{e}},\omega_{\text{p}}\right)$ with high probe and heating power (∼−120dBm) and dividing the subsequent measurements by this high-power reference, that is, $\text{Γ}\left(T_{\text{e}},\omega_{\text{p}}\right)=\tilde{\text{Γ}}\left(T_{\text{e}},\omega_{\text{p}}\right)/\tilde{\text{Γ}}\left(T\gg T_{\text{e}},\omega_{\text{p}}\right)$. The high power shifts the resonance far from its low-power position, thus providing an accurate calibration for the low-power experiments.

### Josephson parametric amplifier.

The utilized JPA is that referred to as Device A by Simbierowicz et al.^42^. It is a lumped-element rf resonator where an array of 200 superconducting quantum interference devices (SQUIDs) forms a non-linear inductor. The SQUIDs are direct current biased and rf pumped with magnetic flux, generating a three-wave mixing process in the JPA. We use the JPA in the non-degenerate mode where the flux pump is at $2\left(f_{\text{p}}+21.875\;\text{kHz}\right)$. Since the pump is at approximately twice the bolometer resonance frequency, we avoid residual heating of the bolometer by the JPA.

### Thermal time constant and electrothermal feedback.

In the low-$P_{\text{p}}$ limit, Γ is independent of the probe power. If the probe power is increased, however, the power $\left(1-{\left|\text{Γ}\left(T_{\text{e}},\omega_{\text{p}}\right)\right|}^{2}\right)P_{\text{p}}$ absorbed from the probe starts to significantly heat the bolometer and shifts the resonance to a lower frequency, as shown in Fig. 1c. More precisely, the nanowire electron temperature $T_{\text{e}}$ is determined by

(2) $$C_{\text{e}}\left(T_{\text{e}}\right){\dot{T}}_{\text{e}}=-P_{\text{e}-\text{b}}\left(T_{\text{e}},T_{\text{b}}\right)+P_{\text{x}}+P_{\text{h}}\;\;+\left(1-{\left|\text{Γ}\left(T_{\text{e}},\omega_{\text{p}}\right)\right|}^{2}\right)P_{\text{p}},$$

if we model the electrons in the nanowire using a single heat capacity $C_{\text{e}}$ and assume that the electrical degrees of freedom relax to a quasistationary state quickly compared to the thermal relaxation time^28^. Here, $P_{\text{e}-\text{b}}\left(T_{\text{e}},T_{\text{b}}\right)$ is the heat flow from the electrons to their thermal environment at temperature $T_{\text{b}}$ and $P_{\text{x}}$ is a constant parasitic heating term arising from uncontrolled noise sources.

Let us analyze the increase in the power flow from the nanowire electrons to their thermal environment, as compared to the case $P_{\text{h}}=P_{\text{p}}=0$. We define this increase as

(3) $$\text{Δ}P\left(T_{\text{e}}\right)=P_{\text{e}-\text{b}}\left(T_{\text{e}},T_{\text{b}}\right)-P_{\text{x}}.$$

It is convenient to discuss ΔP rather than $T_{\text{e}}$ because ΔP can be directly measured^28^ and it allows us to simplify Eq. (2) to

(4) $$\tau_{\text{b}}(\text{Δ}P)\text{Δ}\dot{P}=-\text{Δ}P+P_{\text{h}}+\left(1-{\left|\text{Γ}\left(\text{Δ}P,\omega_{\text{p}}\right)\right|}^{2}\right)P_{\text{p}},$$

where $\tau_{\text{b}}(\text{Δ}P)=C(\text{Δ}P)\partial_{T\text{e}}P_{\text{e}-\text{b}}\left[T_{\text{e}}(\text{Δ}P),T_{\text{b}}\right]$ is the bare thermal time constant^28^, not including the effects of the electrothermal feedback, and $\text{Δ}\dot{P}$ denotes $\partial_{t}\text{Δ}P$.

We concentrate on the non-bistable regime where Eq. (4) has a unique stationary solution. In this regime, we can define the single-valued dimensionless susceptibility given by Eq. (1). The susceptibility also allows us to further simplify Eq. (4) into

(5) $$\chi \left(\omega_{\text{p}},P_{\text{p}}\right)\tau_{\text{b}}\left(\text{Δ}P_{0}\right)\partial_{t}\left(\text{Δ}P-\text{Δ}P_{0}\right)\approx -\left(\text{Δ}P-\text{Δ}P_{0}\right),$$

for small deviations around $\text{Δ}P=\text{Δ}P_{0}$ that solves Eq. (4) in steady state^44^. From Eq. (5) we observe that the effective thermal time constant is given by

$$\tau =\chi \left(\omega_{\text{p}},P_{\text{p}}\right)\tau_{\text{b}}\left(\text{Δ}P_{0}\right).$$

### Noise equivalent power.

We define ${\text{NEP}}^{2}\left(f_{\text{n}}\right)$ of a noisy bolometer as the one-sided power spectral density of input power fluctuations (units: ${\text{W}}^{2}/\text{Hz}$) that yields for an ideal bolometer a noise spectral density in the output signal identical to that of the noisy bolometer. Here, the ideal bolometer refers to a noiseless conversion of input power into output signal with a responsivity equal to that of the noisy bolometer. Equivalently, $2\sqrt{2\text{B}}\times \text{NEP}\left(f_{\text{n}}\right)$ describes, in a narrow bandwidth B centered at $f_{\text{n}}$, the peak-to-peak amplitude by which the input power needs to be modulated at $f_{\text{n}}$ for unit signal-to-noise ratio in the output.

Specifically in this work, the NEP (shown in Fig. 2e–h) is given by

(6) $$\text{NEP}\left(f_{\text{n}}\right)=\frac{\sqrt{S_{\text{V}}\left(f_{n}\right)}}{R_{\text{P}\to V}\left(f_{\text{n}}\right)},$$

where $R_{P\to V}\left(f_{\text{n}}\right)$ is the frequency-dependent responsivity (shown in Fig. 2a, b) and $S_{V}\left(f_{\text{n}}\right)$ is the single-sided power spectral density of the output signal V (Fig. 2c, d shows $\sqrt{S_{V}}$). Note that $\text{B}\times S_{V}\left(f_{\text{n}}\right)$ equals the ensemble variance if the signal is filtered to a narrow bandwidth B centered at $f_{\text{n}}$.

In practice, we measure $S_{V}\left(f_{\text{n}}\right)$ by averaging periodograms according to Bartlett’s method and determine the frequency-dependent responsivity from

(7) $$R_{P\to V}\left(f_{\text{n}}\right)=\frac{\left|\partial_{P_{\text{h}}}V\right|}{\sqrt{1+{\left(2\pi f_{\text{n}}\tau \right)}^{2}}},$$

where $\left|\partial_{P_{\text{h}}}V\right|$ is the measured quasistatic responsivity and τ is the measured time constant. Based on the adequate quality of the fits used to extract τ (see Fig. 1e), this single-time-constant approximation is justified at least up to frequencies of the order of 1/(2πτ). We note that Eqs. (7) and (6) are identical to those used for NEP in the previous literature^33,58^.

### Terahertz simulations.

The simulation tool in analyzing the THz coupling was Ansys Electromagnetic Suite v. 19. The received power of an antenna can be calculated as $P=A_{\text{e}}S$, where $A_{\text{e}}$ is the effective area and S is the power density of an incoming wave. The effective area is $A_{\text{e}}=G\lambda^{2}/4\pi$, where G is the maximum realized gain as a function of frequency, and λ is the corresponding wavelength^59^. The realized antenna gain can be written as $G=\eta_{\text{p}}D$, where $\eta_{\text{p}}$ is the power efficiency, that is, the ratio between the radiated power and the input power, and D is directivity^59^. Here, G includes the loss due to impedance mismatch. The directivity is defined as follows: In a given direction, the part of the radiation intensity corresponding to a given polarization divided by the total radiation intensity averaged over all directions^49^.

## Data Availability

The data that support the findings of this study are available at https://doi.org/10.5281/zenodo.3384598.
